# Impaired lipolysis in propionic acidemia: A new metabolic myopathy?

**DOI:** 10.1002/jmd2.12113

**Published:** 2020-03-31

**Authors:** Jesper H. Storgaard, Karen L. Madsen, Nicoline Løkken, John Vissing, Gerrit van Hall, Allan M. Lund, Mette C. Ørngreen

**Affiliations:** ^1^ Department of Neurology, Copenhagen Neuromuscular Center, Rigshospitalet Copenhagen University Hospital Copenhagen Denmark; ^2^ Department of Biomedical Sciences Rigshospitalet, University of Copenhagen Copenhagen Denmark; ^3^ Department of Clinical Genetics Centre for Inherited Metabolic Diseases, Rigshospitalet, Copenhagen University Hospital Copenhagen Denmark; ^4^ Department of Pediatrics and Adolescent Medicine Centre for Inherited Metabolic Diseases, Rigshospitalet, Copenhagen University Hospital Copenhagen Denmark

**Keywords:** carbohydrate metabolism, exercise metabolism, fat metabolism, metabolic myopathy, organic aciduria, propionic acidemia

## Abstract

The objective of this study was to investigate the fat and carbohydrate metabolism in a patient with propionic acidemia (PA) during exercise by means of indirect calorimetry and stable isotope technique. A 34‐year‐old patient with PA performed a 30‐minute submaximal cycle ergometer test. Data were compared to results from six gender‐ and age‐matched healthy controls. Main findings are that the patient with PA had impaired lipolysis, blunted fatty acid oxidation, compensatory increase in carbohydrate utilization, and low work capacity. Our findings indicate that PA should be added to the list of metabolic myopathies.

**SYNOPSIS**

This study shows impaired energy metabolism in a patient with propionic acidemia indicating that this disease should be considered a metabolic myopathy.

## 1 INTRODUCTION

Propionic acidemia (PA) is a rare organic aciduria caused by a mutation in either the *PCCA* or *PCCB* genes (OMIM numbers *232000 and *232050) causing deficiency of the enzyme propionyl‐CoA carboxylase (PCC).^1^ Propionyl‐CoA is a metabolite arising from the catabolism of the four amino acids valine, threonine, methionine, and isoleucine, as well as odd‐chain fatty acids and cholesterol. PCC catalyses the conversion of propionyl‐CoA to d‐methylmalonyl‐CoA, which in turn is converted to succinyl‐CoA and enters the tricarboxylic acid (TCA) circle.^1^ PA causes an accumulation of alternative metabolites from propionyl‐CoA.^1^

Most patients present with drowsiness and poor feeding within hours to days postpartum. If the metabolic decompensation is not treated, progressive encephalopathy with neurological damage will ensue.^2^ Metabolic crisis often include acidosis and hyperammonaemia.^3^ Approximately, 25% of the patients present after 1 year of age with either symptoms similar to the neonatal onset type or a more chronic, progressive form.^2^

Metabolic decompensation may occur without any apparent reason but is mostly caused by catabolic stress (eg, infection, trauma, surgery, exercise). Treatment of these acute episodes of metabolic decompensation is primarily symptomatic, whereas long‐term prophylactic treatment consists of dietary restriction of propiogenic amino acids, l‐carnitine supplementation and metronidazole.^3^, ^4^, ^5^

In addition to the above‐mentioned symptoms, all the six patients in our clinic with PA and the related disorder methylmalonic aciduria report exercise intolerance. As exercise intolerance has never been investigated in PA, the objective of this study was to examine the metabolism of carbohydrates and fatty acids during exercise in a patient with PA.

## 2 METHODS

### 2.1 Ethical approval

All participants signed an informed consent form. The study was conducted according to the Helsinki Declaration, approved by the Danish National Committee on Health Research Ethics (H‐15015150) and registered at https://clinicaltrials.gov/ (NCT02635269).

### 2.2 Subjects

#### 2.2.1 Patient

A 34‐year‐old woman with biochemically verified PA was included (Table 1). She was diagnosed 2 years old and has been followed closely at our clinic since. She holds a part‐time job and lives a close to normal everyday life without adhering to any strict diet nor any other treatments. She does, however, experience both fatigue and exercise intolerance. The latter presents as stiffness and heaviness of the thigh and calf muscles during exercise. Echocardiography had showed left ventricular hypertrophy and near normal left ventricular ejection fraction at 45%‐50%.

**Table 1:** Age, weight, height, and BMI

Group	Age (years)	Weight (kg)	Height (cm)	BMI	Mean HR during exercise	Mean Borg during exercise	P‐carnitine (μmol L^−1^)
PA patient	34	54.5	153	23	156	13.5	10^a^
Healthy controls	35 ± 9 (23‐46)	62.3 ± 3.8 (58‐69)	169.5 ± 2.8 (166‐174)	21.7 ± 1.4 (21‐24)	85 ± 5.1 (82‐91)	8.0 ± 1.0 (7.0‐9.5)	‐

*Note:* Values for healthy controls are presented as mean ± SD with range in parenthesis.

Abbreviations: BMI, body mass index; PA, propionic acidemia.

a Normal range, 24‐64 μmol L^−1^.

Only one of five patients with PA in our clinic had the sufficient age and level of compliance to participate in the study. This patient was the most mildly affected in our clinic, the other four patients were either unable to perform cycle exercise testing or reported they could not overcome to participate in the study.

#### 2.2.2 Healthy controls

Six healthy gender‐ and age‐matched subjects from a previous study^6^ were included as healthy controls (HC) (Table 1). Methods pertaining to the HC are described in the above‐mentioned study.^6^

### 2.3 Experimental protocol

Stable isotope tracers were prepared as previously described.^7^ The patient came to the laboratory in the morning and received a breakfast meal. Two intravenous catheters were inserted in distal veins in the arms. After blood and air sampling, infusions of [6.6‐^2^H_2_]‐glucose (0.0026 mg kg^−1^ min^−1^, primed with 2.44 mg kg^−1^ (99% enriched, Cambridge Isotope Laboratories, Andover, MA) and [U‐^13^C]‐palmitate (0.0728 mg kg^−1^ min^−1^, primed with 0.085 mg kg^−1^ of NaH_13_CO_3_ [98% enriched, Cambridge Isotope Laboratories, Andover, MA]) were started using a Gemini PC2 Pump (IMED, San Diego, CA). After a basal infusion for 2 hours, the rate was doubled, and the patient performed a 30‐minute submaximal exercise test on a cycle ergometer (Excalibur, Lode, Gronningen, The Netherlands). Heart rate and perceived exertion (Borg score) were noted every second minute.^8^

### 2.4 Sampling and analysis

Samples of blood and expired air were collected before start of infusion and every 10 minutes from 20 minutes before the test started until the test stopped.

Prior to blood sampling, the arm was heated with a heating pad to ensure arterialization of the blood. Blood for measurements of glucose and lactate was sampled in heparinized syringes and analyzed immediately on an ABL90 flex (Radiometer, Bronshoj, Denmark). The remaining blood samples were transferred to vials with ethylenediamine tetraacetic acid and spun at 4°C for 10 minutes. Plasma samples were stored in a −80°C freezer until analysis. Free fatty acids (FFA) were analyzed with Wako NEFA‐HR (2) (Fujifilm Wako Chemicals Europe GmbH, Germany). Plasma carnitine level was analyzed at the Metabolic Laboratory at the Department of Clinical Genetics, Rigshospitalet, Copenhagen.

Air samples were collected in a nondiffusible 15‐L Douglas bag (Hans Rudolph Inc., Shawnee, KS), and transferred to evacuated Exetainer Breath Vials (Labco Limited, Ceredigion, UK). Plasma samples of glucose and palmitate tracer as well as the breath samples were analyzed at Clinical Metabolomics Core Facility, Rigshospitalet, Copenhagen, as previously described.^9^, ^10^

Measurements of gas exchanges were performed with a Cosmed Quark CPET (Cosmed, Milan, Italy).

### 2.5 Calculations and statistical analysis

Tracer calculations were done as previously described.^7^ Fatty acid oxidation (FAO) rates (μmol kg^−1^ min^−1^) were calculated as follows:

$$\left(\left(\left(\frac{\left(1.695*{\mathrm{VO}}_{2}-1.701*{\mathrm{VCO}}_{2}\right)}{860}\;g\;{\mathrm{mol}}^{-1}\right)*1\;000\;000\right)\;{\mathrm{kg}}^{-1}\right)*3.$$

Carbohydrate oxidation (CHO) rates (μmol kg^−1^ min^−1^) were calculated as follows:

$$\left(\left(\frac{4.585\times {\mathrm{VCO}}_{2}-3.226\times {\mathrm{VO}}_{2}}{180}\;g\;{\mathrm{mol}}^{-1}\right)\times 1\;000000\right)\;{\mathrm{kg}}^{-1}$$

Values for HC are presented as mean ± SD.

## 3 RESULTS

Demographic data are described in Table 1. The PA patient had a higher heart rate and Borg score during exercise compared to HC.

### 3.1 Fat and carbohydrate metabolism

**Figure 1:**
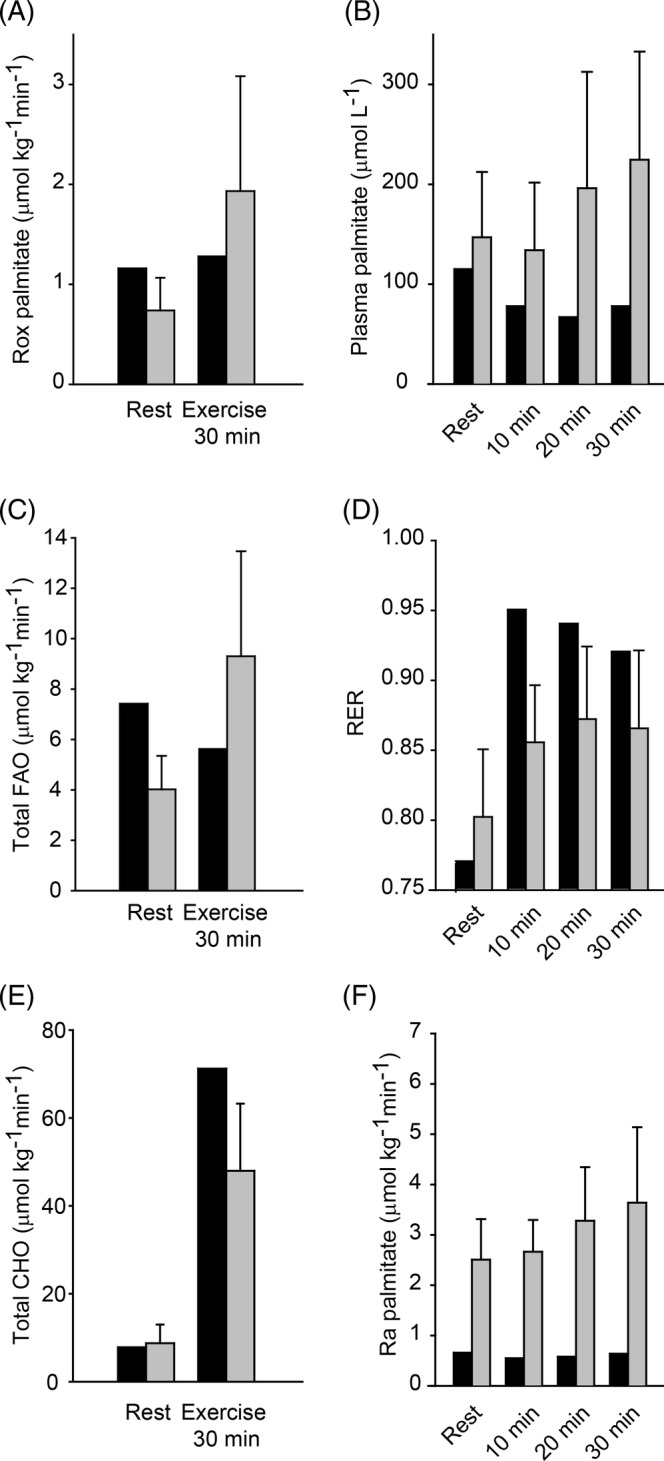
Propionic acidemia (PA) patient (black bars) and six healthy controls (grey bars). Values for healthy controls presented as mean + SD. A, Rate of oxidation (Rox) of palmitate at rest and at 30‐minute exercise. B, Plasma palmitate concentration at rest and during exercise. C, Fatty acid oxidation (FAO) rate at rest and at 30‐minute exercise. D, Respiratory exchange rate (RER) at rest and during exercise. E, Total carbohydrate oxidation (CHO) at rest and at 30‐minute exercise. F, Rate of appearance (Ra) of palmitate at rest and during exercise

FAO and palmitate oxidation were normal at rest, but the normal increase during exercise was blunted in the patient (Figure 1). Furthermore, plasma palmitate and plasma FFA increased during exercise in the HC but not in the PA patient. In line with this, total CHO and respiratory exchange rate (RER) increased to higher values in the PA patient vs HC (Table 2).

**Table 2:** Plasma metabolites, substrate turnover, and gas exchange in the PA patient vs HC

	Patient		Healthy controls	
	Rest	30 min	Rest	30 min
Plasma metabolites				
FFA (μmol L^−1^)	456	380	402 ± 219	518 ± 351
Glycerol (μmol L^−1^)	71	87	140 ± 37^a^	160 ± 47^a^
Alanine (μmol L^−1^)	261	424	260 ± 54^a^	240 ± 51^a^
Palmitate (μmol L^−1^)	115	78	147 ± 65	225 ± 108
Glucose (mmol L^−1^)	4.8	3.8	5.3 ± 0.8	5.1 ± 0.4
Lactate (mmol L^−1^)	0.87	4.74	0.93 ± 0.23^a^	0.76 ± 0.30^b^
Substrate turnover				
Palmitate *R*_a_ (μmol kg^−1^ min^−1^)	0.7	0.6	2.5 ± 0.8	3.6 ± 1.5
Palmitate *R*_d_ (μmol kg^−1^ min^−1^)	0.7	0.6	2.5 ± 0.8	3.5 ± 1.5
Palmitate Rox (μmol kg^−1^ min^−1^)	1.2	1.3	0.7 ± 0.3	1.9 ± 1.2
Total fatty acid Rox (μmol kg^−1^ min^−1^)	7.4	5.6	4.0 ± 1.3	9.3 ± 4.2
Glucose *R*_d_ (μmol kg^−1^ min^−1^)	19.9	28.1	16.3 ± 0.9^a^	14.0 ± 0.3^a^
CHO (μmol kg^−1^ min^−1^)	7.8	71.2	8.7 ± 4.3	48.0 ± 15.2
RER	0.77	0.92	0.80 ± 0.05	0.87 ± 0.06
Gas exchange				
VO_2_ (mL min^−1^)	290	697	217 ± 43	733 ± 79
VCO_2_ (mL min^−1^)	221	643	174 ± 35	633 ± 67

*Note:* Values for healthy controls presented as mean ± SD.

Abbreviations: CHO, carbohydrate oxidation; FFA, free fatty acids; HC, healthy controls; PA, propionic acidemia; *R*_a_, rate of appearance; RER, respiratory exchange rate; *R*_d_, rate of disappearance; Rox, rate of oxidation; VCO_2_, carbon dioxide excretion; VO_2_, oxygen uptake.

a n = 4.

b n = 3.

### 3.2 Plasma metabolites

Plasma lactate levels rose more than fivefold in the PA patient, whereas they remained unchanged in the HC (Table 2). Plasma carnitine was low in the patient. Oxygen uptake was comparable between HC and the patient (Table 1).

## 4 DISCUSSION

The primary objective of this study was to investigate the carbohydrate and fat metabolism during exercise in a patient relatively mildly affected with PA using stable isotope technique and indirect calorimetry. Main findings are that the patient with PA had (a) impaired lipolysis, (b) a blunted FAO, (c) a compensatory increased carbohydrate utilization, and (d) a low work capacity.

Surprisingly, the normal exercise‐induced increase in plasma palmitate and FFA levels was blunted in the PA patient. This impaired lipolysis and lack of substrates for FAO most likely causes the blunted increase in palmitate oxidation and total FAO. Findings are similar to those reported in a patient with neutral lipid storage disease.^11^ However, the lack of substrates might not be the only reason for the blunted FAO in the PA patient, since the plasma carnitine concentration was low, possibly leading to impaired transport of long‐chain fatty acids into the mitochondria for FAO. Carnitine levels are often low in untreated PA patients due to elevated excretion of carnitine bound to organic acids.^4^ Furthermore, a number of in vitro studies indicate that the TCA cycle is impaired due to inhibited enzymes; propionyl‐CoA and its metabolites have been shown to inhibit various mitochondrial enzymes, including pyruvate dehydrogenase complex,^12^ α‐ketoglutarate dehydrogenase complex,^12^ OXPHOS complex III,^12^ succinate‐CoA ligase,^13^ citrate synthase,^14^ aconitase,^14^ and isocitrate dehydrogenase.^14^ This might also contribute to the blunted FAO.

Both total CHO and RER increased to a larger extent in the patient vs HC, indicating that the patient relies more on carbohydrates as energy source than the HC. The patient had a low work capacity as demonstrated by her high mean heart rate of 156 beats per minute during the 30‐minute, low‐level (25 W) constant workload exercise bout. Deconditioning secondary to limited physical activity could influence this poor performance but cannot alone explain this marked reduction in work capacity. A low work capacity has also been observed in a patient with methylmalonic aciduria,^15^ which is a similar disorder caused by a defect in the enzyme methylmalonyl‐CoA mutase situated downstream from PCC, suggesting a mutual pathophysiology pertaining to the low workload. Another clinically comparable patient group are patients with McArdle disease (glycogen storage disease V), who also have a low workload capacity.^16^, ^17^ They have an impaired or most often completely blocked breakdown of muscular glycogen.^18^ During exercise, the FAO in McArdle patients initially rise to compensate for the lack of energy‐substrates from muscular glycogen. Though the plasma FFA levels increase throughout exercise, the FAO rate remains at the level achieved during the first part of exercise and is unable to fully compensate for the energy shortage^6^ This is probably due to lack of substrates in the TCA cycle^6^, ^19^ which together with the blocked glycogenolysis results in the low oxidative capacity and work capacity. A possible explanation for the low work capacity in the PA patient could be a similar shortage of substrates in the TCA cycle.

In conclusion, we found impaired lipolysis, blunted FAO, elevated carbohydrate utilization, and low work capacity in a patient relatively mildly affected by PA. Same symptoms characterise our other more severely affected patients with PA, and our results suggest that PA should be considered a metabolic myopathy. However, further investigations are required to confirm these novel findings of perturbed fat metabolism in both mildly and more severely affected patients with PA.

## CONFLICT OF INTEREST

The authors declare no potential conflict of interest.

## AUTHOR CONTRIBUTIONS

A.M.L. and M.C.Ø.: conception and design of the study; K.M.L., N.L., M.C.Ø., G.V.H., J.H.S., and J.V.: acquisition and analysis of data; J.H.S.: drafting of manuscript; A.M.L., M.C.Ø., K.M.L., N.L., G.V.H., J.V., and J.H.S.: reviewing and approval of manuscript. J.H.S.: author serving as guarantor for the article.

## Data Availability

Due to the Danish Data Protection law, no data from this study can be shared with a third party until the study subject ID list is destructed on December 31, 2025. In urgent cases, a special application can be submitted to The Danish Data Protection Agency, Regional Scientific Ethic Committee or the Copenhagen Region Denmark.
