# Corrigendum

**DOI:** 10.1002/jmd2.12257

**Published:** 2021-11-08

In the following article,^1^ the infusion rates stated in section 2.3 Experimental Protocol, are misplaced in forth sentence. The correct rates should read as follows:

After blood and air sampling, infusions of [6.6‐^2^H_2_]‐glucose (**0.0728** mg kg^−1^ min^−1^, primed with 2.44 mg kg^−1^ (99% enriched, Cambridge Isotope Laboratories, Andover, MA) and [U‐^13^C]‐palmitate (**0.0026** mg kg^−1^ min^−1^, primed with 0.085 mg kg^−1^ of NaH_13_CO_3_ [98% enriched, Cambridge Isotope Laboratories, Andover, MA]) were started using a Gemini PC2 Pump (IMED, San Diego, CA).

The authors apologise for the errors.
